# Genetic diversity and evolutionary insights of respiratory syncytial virus A ON1 genotype: global and local transmission dynamics

**DOI:** 10.1038/srep14268

**Published:** 2015-09-30

**Authors:** Venkata R. Duvvuri, Andrea Granados, Paul Rosenfeld, Justin Bahl, Alireza Eshaghi, Jonathan B. Gubbay

**Affiliations:** 1 Public Health Ontario, Toronto, Ontario, Canada; 2 University of Waterloo, Waterloo, Ontario, Canada (MPH student); 3 University of Toronto, Ontario, Canada; 4 Center for Infectious Diseases, The University of Texas School of Public Health, Houston, Texas, United States of America; 5 Mount Sinai Hospital, Toronto, Ontario, Canada; 6 The Hospital for Sick Children, Toronto, Ontario, Canada

## Abstract

Human respiratory syncytial virus (RSV) A ON1 genotype, first detected in 2010 in Ontario, Canada, has been documented in 21 countries to date. This study investigated persistence and transmission dynamics of ON1 by grouping 406 randomly selected RSV-positive specimens submitted to Public Health Ontario from August 2011 to August 2012; RSV-A-positive specimens were genotyped. We identified 370 RSV-A (181 NA1, 135 NA2, 51 ON1 3 GA5) and 36 RSV-B positive specimens. We aligned time-stamped second hypervariable region (330 bp) of G-gene sequence data (global, n = 483; and Ontario, n = 60) to evaluate transmission dynamics. Global data suggests that the most recent common ancestor of ON1 emerged during the 2008–2009 season. Mean evolutionary rate of the global ON1 was 4.10 × 10^−3^ substitutions/site/year (95% BCI 3.1–5.0 × 10^−3^), not significantly different to that of Ontario ON1. The estimated mean reproductive number (*R*_*0*_ = ∼ 1.01) from global and Ontario sequences showed no significant difference and implies stability among global RSV-A ON1. This study suggests that local epidemics exhibit similar underlying evolutionary and epidemiological dynamics to that of the persistent global RSV-A ON1 population. These findings underscore the importance of continual molecular surveillance of RSV in order to gain a better understanding of epidemics.

Human Respiratory Syncytial Virus (RSV) is the most common cause of severe lower respiratory tract infection (LRTI) in infants and young children, resulting in 100,000 hospitalizations per year in the USA from pneumonia and bronchiolitis^1^. By the age of two, nearly all children have been infected with RSV^2^. In adults, RSV infections usually range from asymptomatic to moderately severe upper respiratory tract presentations^3^. In the elderly, RSV causes exacerbations of COPD, acute deterioration of cardiac disease, and deaths in the winter season^2^. Currently, there is no effective treatment or vaccine available^4^.

RSV is an enveloped virus with a negative sense, single-stranded RNA genome of approximately 15,000 nucleotides that is classified in the Pneumovirus genus of the *Paramyxoviridae* family. The viral genome encodes 11 proteins. Of these, the G- and F- proteins are the major surface antigens of RSV which are involved in virus attachment to cell receptors and the mediation of cell membrane fusion, respectively^5^,^6^. Both G- and F- proteins are accessible to neutralizing antibodies, however only the G-protein is known to accumulate mutations in response to host immunological pressures^7^.

RSV is classified into two groups, RSV-A and RSV-B, on the basis of reactions with monoclonal antibodies against surface antigens^8^. RSV-A and RSV-B viruses subdivide into 12 genotypes [GA1-GA7, SAA1, NA1–2 and ON1–2] and 20 genotypes [GB1–4, BA1–10, SAB1–4, and URU1–2], respectively, based on the genetic variability of the G-protein gene^9^,^10^,^11^,^12^. The RSV-A ON1 genotype was first detected in November, 2010 in Ontario, Canada^11^, and subsequently a retrospective study (2008–2012) from Panama found RSV-A ON1 in a sample collected in October, 2010 (accession number: KF300973). The RSV-A ON1 signature is a tandem repeat of 72 nucleotides (corresponding to 24 amino acid residues) in the C-terminal region of the G-protein^11^. Interestingly, BA genotype strains (first detected in 1999) of RSV-B from Buenos Aires, Argentina exhibited a 60 nucleotide duplication in the second variable region of the G protein gene, and became established globally with different lineages (BA1 to BA10)^10^. In 2014, Hirano *et al.*^12^ reported that there are three lineages of RSV-A ON1 circulating globally. In 2014, Hirano et al.12 reported that there are three lineages of RSV-A ON1 circulating globally and a possible new genotype RSV-A ON2 in Rome, Italy in 2013. This G-gene diversity, with mean evolutionary rates 2.22 × 10^−3^ for RSV-A and 2.78 × 10^−3^ for RSV-B of RSV strains may alter the virus pathogenicity, fitness, and the ability of RSV to establish reinfections throughout life^1^. As of November 14 2014, available literature and NCBI’s GenBank sequence database confirms that RSV-A ON1 has been documented in 21 countries^4^,^9^,^11^,^12^,^13^,^14^,^15^,^16^,^17^,^18^,^19^,^20^,^21^,^22^,^23^,^24^,^25^,^26^.

In this study we investigate the genetic diversity, lineage distribution, time of the most recent common ancestor (tMRCA), and basic reproductive number (*R*_*0*_) for RSV-A ON1 genotype through comparative analyses of G-gene sequences from a global dataset. This global dataset consisting of all available data in NCBI’s GenBank sequence database (n = 483, 330 bp region; Set G330) was compared with a representative dataset consisting of 293 sequences after the removal of identical sequences originating in the same country (Set G330R), and a 696bp region (n = 281; Set G696) covering four RSV seasons (2010–2014). Finally a dataset consisting of ON1 sequences from local specimens in Ontario, Canada (n = 60, 330bp region; Set ON) was characterized and compared with global (including Ontario) populations to investigate the underlying evolutionary and transmission dynamics of RSV-A ON1.

## Results

### Population demographics and RSV-A distribution in Ontario

**Figure 1:**
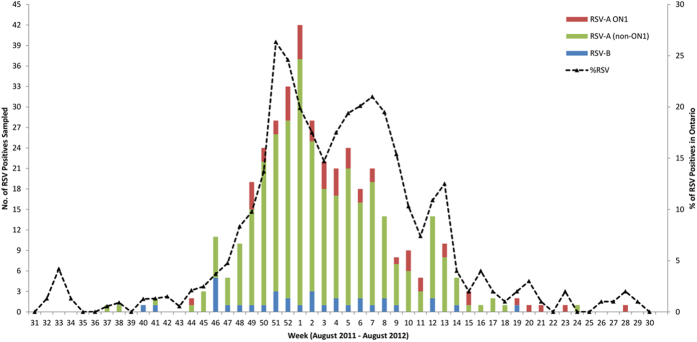
Circulating trends of RSV in Ontario, Canada. The weekly distribution of RSV-A and -B among the randomly selected sample set (columns) and the percent positivity of RSV-A and -B among all respiratory specimens tested at Public Health Ontario (dotted lines). Percent positivity data obtained from the Public Health Ontario Laboratory-based Respiratory Pathogen Surveillance Report: week 51–52 (December 18–31, 2012) and week 30–31 (July 22-August 4, 2012)^60^.

Between August 2011 and August 2012, 2101 RSV-positive samples were identified at Public Health Ontario (PHO). Of these, we randomly selected 406 samples. RSV-A (370/406, 91.1%) was the most common group circulating in Ontario whereas RSV-B was identified in 36 (8.9%) samples. The temporal prevalence of RSV-positive samples included in the study and the percent positivity of RSV among all respiratory specimens tested at PHO per calendar week is shown in Fig. 1. Among these 406 RSV-positive specimens, the majority (240/406; 59.1%) were submitted from children <1 year old. Females were more often affected than males by ON1 (30/51, 58.8% female, χ^2^ = 6.69, p = 0.0097) (Table 1). G-gene sequencing of the 370 RSV-A-positive specimens identified 4 RSV-A genotypes circulating in Ontario: NA1 (n = 181, 48.9%), NA2 (n = 135, 36.4%), ON1 (n = 51, 13.7%), and GA5 (n = 3, 0.8%).

### Global distribution of RSV-A ON1 genotype

**Figure 2:**
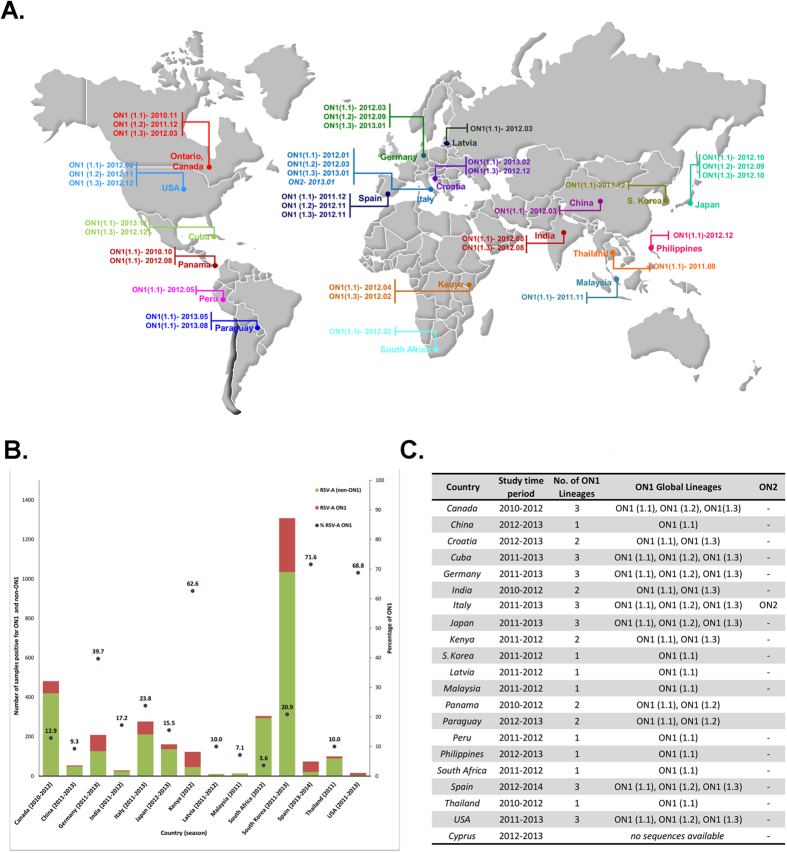
(**A**) Spread of RSV-A ON1 as of 14 November 2014, (**B**) ON1 prevalence among other RSV-A genotypes isolated in different regions^#^, and (**C**) global ON1 lineage distribution. All data were collected from the published literature^4^,^9^,^11^,^12^,^13^,^14^,^15^,^16^,^17^,^18^,^19^,^20^,^21^,^22^,^23^,^24^,^25^,^26^. ^#^Due to sampling bias, data provided here may not be reflective of true country-wise prevalence rates. The exact geographical area of specimen collection from each country is tabulated in Table S2. The freely editable vector map of the world template was downloaded from presentationmagazine.com (http://www.presentationmagazine.com/world-maps-vector-editable-507.htm). The map was created with PowerPoint and Adobe Photoshop.

The geographic distribution of RSV-A ON1 along with date of detection of each ON1 lineage was mapped based on the place of isolation as documented in GenBank as of 14 November 2014 (Fig. 2A). Twenty one countries (Canada, China, Croatia, Cuba, Cyprus, Germany, India, Italy, Japan, Kenya, South Korea, Latvia, Malaysia, Panama, Paraguay, Peru, Philippines, South Africa, Spain, Thailand, and USA) reported circulation of RSV-A ON1 during 2010–2014 (Table S1, Table S2). RSV-A ON1 prevalence compared to other RSV-A genotypes in 14 countries is presented in Fig. 2B. This information was compiled by summarizing the data provided in published literature^4^,^9^,^11^,^12^,^13^,^14^,^15^,^16^,^17^,^18^,^19^,^20^,^21^,^22^,^23^,^24^,^25^,^26^. Prevalence rates of RSV-A ON1 are reported from Kenya, Spain and USA with ranges of 62.6% to 71.6% followed by Germany, Italy, and South Korea (20.9% to 39.7%), Canada, India, Japan, Latvia, and Thailand (10% to 17.2%) and China, Malaysia and South Africa (3.6% to 9.3%).

### Phylogenetic analysis of RSV-A ON1 genotype

**Figure 3:**
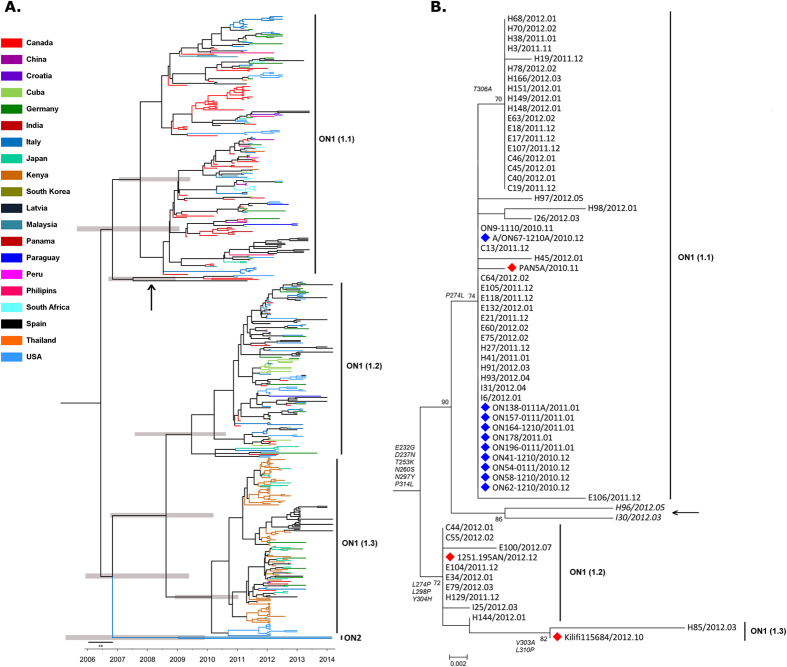
(**A**) Circulating lineages of RSV-A ON1 genotype globally during 2010 to 2014, and (**B**) Ontario during 2010 to 2012. (**A**) Maximum Clade Credibility tree of global RSV-A ON1 G-gene sequences constructed by the Bayesian Markov chain Monte Carlo (MCMC) method^34^. The tree is annotated with midpoint rooting using Figtree (http://tree.bio.ed.ac.uk/software/figtree/). A possible new emerging lineage (cluster) is identified with a black arrow. Light grey shaded bars represent the 95% Bayesian Credible Interval (BCI). (**B**) ON1 sequences collected during the previous Ontario study (2010–2011 season) are marked with a blue diamond. The red diamond indicates the identifier for each lineage. A possible new emerging lineage is identified with black arrow. Multiple sequences alignment and phylogenetic trees were constructed using Clustal W and neighbour-joining algorithm using the Maximum Composite Likelihood (MCL) approach running within MEGA 6.0 software^53^. Tree topology was supported by bootstrap analysis with 1000 pseudo replicate datasets. Bootstrap values greater than 70 are shown at the branch nodes.

The Maximum Clade Credibility (MCC) tree revealed three different RSV-A ON1 lineages circulating globally, indicated as ON1 (1.1), ON1 (1.2), ON1 (1.3), and the recently reported genotype, ON2 (Fig. 3A)^12^. Figure 2C describes the circulating lineages by country. All three global lineages, ON1 (1.1), ON1 (1.2), ON1 (1.3) are co-circulating in seven (Canada, Cuba, Germany, Italy, Japan, Spain and U.S.A) of the 20 countries that have reported ON1 to date. ON1 (1.1) and ON1 (1.3) are co-circulating in Croatia, India, and Kenya; and ON1 (1.1) and ON1 (1.2) are co-circulating in Panama and Paraguay while the remaining eight countries only documented ON1 (1.1) circulation. Based on its genetic divergence (p-distance 0.0072), RSV-A ON2, recently identified by Hirano *et al.* (2014), is currently emerging in Italy^12^.

A phylogenetic tree of Ontario’s RSV-A ON1 sequences (Set ON) is presented in Fig. 3B.

### Phylodynamic history of RSV-ON1 genotype

Table 2 presents the mean global estimates of evolutionary rates (substitution/site/year), tMRCA and basic reproductive number (*R*_*0*_) derived from logistic growth and exponential growth coalescent models implemented in BEAST analyses with different G-gene sequence Sets, G330, G330R and G696. The mean global tMRCA estimates are shown to be similar within 330 bp length sequences as 2008.08 (Set G330) and 2008.81 (Set G330R), as well as Set G696 gave a tMRCA of 2007.77 (Table 2). The Path-O-Gen^27^ (root-to-tip genetic divergence on the ML trees) estimated 2008.95 (Set G330), 2008.72 (Set G330R) and 2007.67 (Set G696) (Table 3). The tMRCA estimates from both methods have considerable overlap. Table 3 reports the comparative mean global tMRCA estimates of RSV-A ON1 along with the models employed and sample sizes. Divergence time estimates using the Ontario dataset resulted in a tMRCA of 2009.70 (95% BCI 2007.98 to 2010.53) with exponential growth model, and 2009.46 (95% BCI 2007.37 to 2010.51) with the logistic growth model (Table 2). The Path-O-Gen^27^ estimated 2009.59 (Set ON).

The mean global evolutionary rate of RSV-A ON1 is estimated to be similar with both population growth models: exponential [4.1 × 10^−3^ substitution/site/year (95% BCI 3.1 × 10^−3^ to 5.0 × 10^−3^) with Set G330 and 4.12 × 10^−3^ substitution/site/year (95% BCI 2.3 × 10^−3^ to 5.4 × 10^−3^) with Set G330R] and logistic [4.02 × 10^−3^ substitution/site/year (95% BCI 3.04 × 10^−3^ to 5.04 × 10^−3^) with Set G330 and 4.0 × 10^−3^ substitution/site/year (95% BCI 2.5 × 10^−3^ to 5.03 × 10^−3^) with Set G330R]. With Set G696, the global evolutionary rate was estimated to be 2.4 × 10^−3^ substitution/site/year (95% BCI 1.8 × 10^−3^ to 3.07 × 10^−3^) (Table 2). The evolutionary rate in each country for which adequate sequence data was available is presented in Table S3. The RSV-A ON1 sequences from Italy, Germany and Japan showed highest mean evolutionary rates, 4.04 × 10^−3^ substitution/site/year, 5.5 × 10^−3^ substitution/site/year, 6.6 × 10^−3^ substitution/site/year, respectively when compared with Ontario, Canada (3.12 × 10^−3^ substitution/site/year), Kenya (2.23 × 10^−3^ substitution/site/year), Spain (1.56 × 10^−3^ substitution/site/year), Panama (2.97 × 10^−3^ substitution/site/year) and the USA (1.9 × 10^−3^ substitution/site/year). Rate estimates from each country had overlapping credible intervals suggesting no significant differences (95% BCI 1.0 × 10^−3^ to 6.6 × 10^−3^).

### Basic reproductive number (*R*_*0*_) estimation from RSV-A ON1 G-gene sequences

We estimated the average *R*_*0*_ of the global population using the estimated growth rates (r in years) of RSV-A ON1 genotype from the population growth models: exponential [r = 1.01, 95% BCI 0.63 to 1.37 with Set G330; r = 1.7, 95% BCI 1.2 to 2.3 with Set G330R; and r = 0.715, 95% BCI 0.42 to 1.01 with Set G696] and logistic [r = 0.86, 95% BCI 0.46–1.33 with Set G330; r = 1.69, 95% BCI 1.02 to 2.2 with Set G330R; and r = 0.89, 95% BCI 0.12 to 1.78 with Set G696] and estimated mean serial interval of RSV^28^, the potential *R*_*0*_ of RSV-A ON1 was determined. The estimates of *R*_*0*_ were obtained using *R*_*0*_* = *(1 + r/b)^a (see Methods). Table 2 presents the mean global *R*_*0*_ values of RSV-A ON1. Both exponential and logistic models with different global datasets from multiple outbreaks derived *R*_*0*_ values just barely exceeded 1.0 i.e., 1.03 (1.02 to 1.04). The mean *R*_*0*_ estimate for Ontario is 1.03 (1.007–1.07). We found similar mean *R*_*0*_ estimate from all other countries (Table S3). This estimate was averaged from reconstructed genealogies including geographically separated detections of ON1 across multiple epidemic seasons. Therefore, the BCI of the *R*_*0*_ estimate can be interpreted as a minimum bound for the recurring circulation of ON1 and where the global population is stable.

### Selection pressure analysis

Relative contributions of evolutionary selection forces on the C-terminal hypervariable region of the G-gene of ON1 globally and in Ontario were separately assessed by measuring the site-specific dN/dS ratio using PAML^29^ (Table S5). ON67-1210A (accession number: JN257693) was used as the reference strain in both analyses. The mean dN/dS ranged from 0.66 to 1.30 and 0.65 to 1.23 among all null and alternative models among global and Ontario ON1 strains, respectively. In both cases, we observed M2a and M8 models provide significant fit with different datasets as evaluated by likelihood ratio tests (LRT = 2Δ*l*) than do their counterpart models, M1a and M7 respectively. Both positive selected models (M2a and M8) suggested the evidence of positively selected sites (PSS) with a proportion ranging from 25% (*p1 = 0.25* with ω = 3.24 from M8) to 29% (*p2* = *0.29* with ω = 2.93 from M2a) with global sequences (Set G330), and 15% [*p1* = *0.15* with 6.43 (M8), *p2* = *0.15* with ω = 6.42 (M2a)] PSS with Ontario sequences (set ON).

A total of 41 amino acids (AA) and 12 AA with posterior probability (PP) greater than 50% were observed among global sequences (Set G330) and Ontario sequences (set ON), respectively. Seven AAs (225, 232, 247, 274, 303, 304 and 318) among global and one AA (303) among Ontario sequences were identified as being under positive selection with a 90% confidence level (Table S6). Table S6 lists other AAs that have >50% to <70% PP, and >70% to <90% PP. Seven AA (225, 273, 274, 289, 306, 310 and 319) are commonly found between global and Ontario RSV-A ON1 sequences. AAs 241, 303 and 312 are unique in Ontario sequences. The following PSSs correspond to each ON1 lineage based on Ontario phylogeny: ON1 (1.1): P274L, T306A; ON1 (1.2): P274L, Y304H; ON1 (1.3): V303A, L310P.

## Discussion

From our sample set of RSV-positive specimens we observed that the majority of RSV-A and B infections occurred during winter and in children less than one year of age. This is consistent with previous reports of RSV seasonality and decreasing incidence with increasing age^30^. Interestingly, we found that ON1 was more likely to be detected in females than males in our population (p = 0.0097). Pierangeli *et al.*^19^ and Tabatabai *et al.*^25^ have reported that males represent 60% of persons with ON1 in their study populations; however they did not detect a significant sex difference because their study populations consistently had more males. As far as we know, a female predominance has not been reported elsewhere. Unfortunately, we did not have clinical data available to determine whether RSV-A ON1 was more or less virulent than other RSV genotypes. We did sort the Ontario RSV-positive samples according to patient settings which may reflect severity of illness [i.e. community acquired infections, emergency room, hospitalized (non-ICU) and intensive care unit (ICU)] and there was no significant difference in the distribution of RSV genotypes. Future studies should include clinical data in order to compare virulence of these emerging genotypes.

In the current study we observed the circulation of four RSV-A genotypes: NA1 (48.9% of RSV-A), NA2 (36.4% of RSV-A), GA5 (0.8% of RSV-A) and ON1 (13.7% of RSV-A) in Ontario during 2011–2012. When we compare with the RSV-A genotypes documented during the 2010–2011 RSV season in Ontario, NA1 (89% of RSV-A) dominated in 2010–2011, followed by ON1 (10% of RSV-A) and GA5 (1.8% of RSV-A). This consecutive molecular surveillance confirms that there has been a genotype shift in Ontario, with NA2 being absent in 2010–11 and increasing to represent almost 40% of all RSV-A positive genotyped specimens. Our findings differ from the earliest Canadian study, which documented a high prevalence of GA5 and GA7 genotypes among Winnipeg isolates in 2000, with each accounting for 30% of circulating RSV-A isolates at that time^31^. Although RSV-A ON1 prevalence increased from 9.8% (11/112) to 13.7% (51/370) between 2010–11 and 2011–12 RSV seasons in our two Ontario studies, this was not a significant rise (p = 0.11).

Since the first detection of RSV-A ON1 in Ontario, Canada in 2010, RSV-A ON1 prevalence has remained relatively stable at 13%^11^ (Fig. 2C). However, there are currently no other Canadian reports on the prevalence of RSV-A ON1 outside of Ontario. This is most likely due to limited RSV molecular surveillance in Canada. The increase in global RSV surveillance has demonstrated that the RSV-A ON1 genotype is disseminating and diversifying with different lineages, as well as the emergence of a new genotype, ON2. The highest prevalence rates occur in Kenya, Spain and the USA (62.6%–71.6%)^13^,^14^,^26^. However, this data was compiled using only published literature, and should not be applied to each country as a whole and may underestimate or overestimate the prevalence of RSV-A ON1 (Table S2).

The global phylogeny represents the widespread occurrence and increasing prevalence of the RSV-A ON1 genotype during four RSV seasons (2010–2014) (Fig. 3A). These trees delineate the circulation of three different lineages [(ON1 (1.1), ON1 (1.2), ON1 (1.3), and the recently described genotype ON2]. In addition to the co-circulation of all three global ON1 lineages in Ontario, Canada [(2010–2011 season: ON1 (1.1), 2011–2012: ON1 (1.1), ON1 (1.2), and ON1 (1.3)], two of Ontario’s 2011–2012 sequences (I30/2012.03, H96/2012.05) clustered separately with a significant bootstrap value (86%) and p-distance value (0.0248). This newly formed cluster could establish a new lineage in Ontario. Interestingly, these sequences formed a small cluster along with sequences from USA (USA/LA2_55), Thailand (CU2011/112) and Italy (1251-.94RM) within ON1 (1.1) lineage on global phylogeny (Fig. 3A). The presence of all three ON1 lineages in 2011–2012 season [after only ON1 (1.1) was found during 2010–2011 season], including the newly formed lineage may suggest the occurrence of multiple introductions into Ontario. The detection of ON1 (1.1) during the consecutive 2010–11 and 2011–2012 seasons suggests that there is continual transmission from the previous season, however follow-up surveillance in Ontario may further describe the local transmission dynamics and persistence.

The phylogenetic analysis revealed the distribution of different ON1 lineages globally (Fig. 2C). Most countries that have conducted RSV surveillance for two consecutive seasons reported the circulation of all three lineages, hence we cannot completely rule out the effect of study time period on the data provided in Fig. 2C. Despite the short or long temporal span of different surveillance studies, all countries reported the circulation of ON1 (1.1) lineage, hence we hypothesize that ON1 (1.1) was more widespread than other lineages. The tMRCA and earlier reports of ON1 (1.1) suggest that it emerged prior to the other ON1 lineages, and therefore it would have had more time to spread globally. This global spread of RSV-A ON1 may also be indicative of travel associated respiratory illness^32^,^33^, or a selective fitness advantage. Future surveillance studies should place an emphasis on recent travel to track the spread of respiratory viruses.

Our global tMRCA analyses with different G-gene sequence sets, G330, G330R and G696 estimated similar tMRCAs. The tMRCA estimate obtained from Set G330 (the two ON2 sequences were omitted for tMRCA analysis) indicates that RSV-A ON1 possibly emerged during the 2007–2008 or 2008–2009 RSV seasons [2008.08 (2006.46–2009.42) by BEAST^34^ and September 2008 by Path-O-Gen^27^] (Table 3). This point estimate is in agreement with the estimate reported by Agoti *et al.* (tMRCA: 2008.8)^14^. We also observed that the tMRCA estimates with sets G330R and G696 differed at almost one year with the tMRCA estimate of Set G330, demonstrating that estimates can be influenced by the number and length of sequences used in analyses (Table 3). Removal of the ON2 outlier sequences from the analysis might be key to confidently estimate meaningful tMRCA as this is a different genotype than ON1. A recent tMRCA estimate for ON1 of 2005 (2000–2010)^12^ may be a result of limited sequence availability (93 ON1 sequences) as indicated by the greater uncertainty in estimates than presented previously^14^ and in the current study here.

Our Bayesian MCMC analyses with different global datasets allowed us to investigate the differences in the mean evolutionary rates (substitutions/site/year). We observed a higher rate of evolution with Set G330 (4.12 × 10^−3^ [95% BCI 2.3 × 10^−3^ to 5.4 × 10^−3^]) than Set G696 (2.4 × 10^−3^ [95% BCI 1.8 × 10^−3^ to 3.07 × 10^−3^]) (Table 2). The global estimate from our study of 4.12 × 10^−3^ (Set G330) is within the ranges of previously predicted ON1 evolutionary rates [5.27 × 10^−3^ (95% BCI 1.53 × 10^−3^ to 9.11 × 10^−3^)^14^ and 6.03 × 10^−3^ (95% BCI 3.43 × 10^−3^ to 9.10 × 10^−3^)^12^]. Our ON1 evolutionary rate 4.12 × 10^−3^ is almost similar to the rate of BA genotype, 4.7 × 10^−3^, estimated using the 330bp of the second hypervariable region^10^. We obtained a lower rate of evolution (2.4 × 10^−3^) with Set G696 than Sets G330 and G330R. This finding is in keeping with previous knowledge that the 330bp 2^nd^ hypervariable region of the G protein is under greater selection pressure than the rest of the G protein, as this is the main target for antibody binding. This finding may also be an artifact of sampling bias as there were no 696bp length sequences available from the following countries reporting high activity of ON1 and increased surveillance: Canada (2011–2012), Germany (2011–2013), Japan (2011–2013), and Italy (2011–2013). However, it would be worth conducting a comparative study using whole genomic and complete G-genes to investigate the RSV-A ON1 evolution. Our study focused on the hypervariable region which is known to mutate at a faster rate than the rest of the G gene, which may result in increased uncertainty in estimates. A previous study by Tan *et al.*^7^ reported lower estimate of evolutionary rate with the whole RSV-A genome (6.47 × 10^−4^) than RSV-A G gene (22.2 × 10^−4^) sequences. The comparative analyses of rate of ON1 evolution between countries showed evidence of higher mutation rates in Italy (4.04 × 10^−3^), Germany (5.5 × 10^−3^) and Japan (6.6 × 10^−3^) than in Ontario (3.12 × 10^−3^). These evolutionary differences of ON1 at a local level could reflect the influence of local host population contact structures and immunological differences.

The site-specific evolutionary analysis revealed strong evolutionary selection pressure i.e., mean dN/dS = 6.43, and mean dN/dS = 3.24 with global (Set G330) and Ontario (Set ON), respectively. A total of 41 AAs (Set G330) and 20 AAs (Set ON) were under selection pressure compared to the ON1 reference strain, ON67-1210A (accession number: JN257693). The difference in total number of AAs may be due to availability of larger data set with global than Ontario. This strong positive selection pressure can be explained by the high evolutionary nature of C-terminal hypervariable region of G-gene, which contains multiple epitopes recognized by both murine monoclonal antibodies (MAbs) and human convalescent sera^35^. Four AAs (225, 226, 274, and 290) of 41 PSS among all global ON1 lineages, and two AAs (225, and 274) of 20 PSS among Ontario ON1 lineages were previously described as escape mutants selected with specific MAbs^36^,^37^. We also observed that AAs 233, 260, 274 and 290 exhibited “flip-flop” pattern when compared with the prototype RSV-A2 and these reversible mutations may decrease the antigen avidity to the current circulating strain specific antibodies^11^,^38^. Similar “flip-flop” patterns of AAs were also reported in non-ON1 RSV-A genotypes^36^,^38^. Interestingly, AA 225 is found to be an escape mutant of the RSV-A Long strain selected with group-specific MAb L9, which can neutralize both RSV-A and RSV-B strains^39^. Substitutions at AAs 226 and 290 resulted in the loss of group-specific, and AA 274 in loss of strain-specific epitopes, respectively^37^,^40^. Less is known about the effects of AA replacements at other sites, (232, 246, 247, 248, 249, 250, 251, 262, 266, 272, and 292) although they were located at antigenic sites^36^,^38^, and 249 AA is close to an antigenic site (250–258)^41^.

Basic reproductive number (*R*_*0*_) is an important index in epidemiology that helps to predict the spread of an infection or vector^42^,^43^. A mathematical modelling analysis on RSV estimated *R*_*0*_ ranged from 1.2 to 2.1^44^. We estimated a global mean *R*_*0*_ between 1.013 (1.008–1.026) and 1.017 (1.012–1.022) for Set G330, 1.013 (1.008–1.018) and 1.032 (1.023–1.047) for Set G330R, and 1.011 (1.002–1.021) and 1.013 (1.008–1.018) for Set G696 across multiple epidemics during 2010–2014 in twenty countries. This averaged value close to 1.0 indicates that the population is no longer in an exponential growth phase and is stable in the human population. Our estimate assumes mean generation time of 7 days (SD: 3.5 days) for converting growth rate (r) to *R*_*0*_. However, this may be insufficient as limited information on serial interval is available. Furthermore, our estimates are averaged over multiple seasons. We assume the prevalence is similar in all countries and all seasons. Our country-wise (cumulative season-based data) *R*_*0*_ findings indicate that the chain of transmission is self-sustaining and stable (Table S3). Multiple stable populations may be a requirement for a persistent globally distributed meta-population. This finding is supported by the establishment of RSV-A ON1 in several countries with an indication of RSV-A genotype shifting and increasing prevalence of RSV-A ON1 (Fig. 2B). While our *R*_*0*_ estimates are lower than those estimated for other diseases, this is likely an artefact of the assumptions and incomplete sampling of multiple epidemics. Further epidemiological and molecular surveillance with matching reports of community prevalence will improve future estimates. Holmes (2008) pointed out that the quality of any inference of population dynamics will be largely affected by the timing and design of sampling protocols^45^,^46^. Various authors have utilized genetic information (genes, and genomes) of different pathogens to estimate the *R*_*0*_ to assess the risk of spread and to understand epidemic behaviors (Table S4). The sequence-based *R*_*0*_ estimates on pandemic 2009 H1N1, 1.2^47^ and 1.12^48^, support previous *R*_*0*_ estimates, 1.3–1.7, from incidence data^47^,^48^ but are close to the lower end of incidence data estimates. Surprisingly, with the lower *R*_*0*_ estimates (even from incidence data), pandemic 2009 H1N1 virus exhibited rapid global spread with its short generation time (2.6 ± 1.3 days) and caused multiple outbreaks^47^,^48^. The trend of low *R*_*0*_ and rapid spread of RSV-A ON1 is likely comparable to that observed for pandemic 2009 H1N1. However, further research is needed to substantiate this observation.

In conclusion, RSV-A ON1 is evolving and disseminating quickly throughout the world with different ON1 lineages and has already diverged into a distinct genotype, ON2. Taken together these results suggest that local epidemics exhibit similar underlying evolutionary and epidemiological dynamics to that of the persistent global RSV population. Linking both epidemiologic and genetic data will improve future estimates and allow for real-time characterization of molecular epidemiology of infectious diseases. To gain a better understanding of this enhanced biologic fitness, we are currently conducting whole genome sequencing on selected ON1 samples from the Ontario population. Continual surveillance of emerging respiratory viruses is necessary to gain a better understanding of their epidemic potential, as well as for the development of targeted therapies.

## Methods

### Ethics

This study was approved by the Public Health Ontario (PHO) Research Ethics Board and was considered exempt from University of Toronto’s Health Sciences Research Ethics Board review as it involved de-identified respiratory tract samples that were tested as part of routine clinical virology services provided by PHO. Methods were carried out in accordance with guidelines approved by the PHO Research Ethics Board. Samples and isolates included in this study were analyzed as part of PHO’s respiratory viral molecular surveillance program that supports Ontario’s Ministry of Health and Long-Term Care.

### Specimen collection

PHO performs a large proportion of primary respiratory viral testing for a variety of clinical settings including clinics, hospitals and outbreaks in the province of Ontario. A random sampling of RSV positives from August 2011 to August 2012 was selected (n = 406). Nasopharyngeal swabs (NPS) from emergency rooms, hospitalized in-patients and clinics are cultured for virus isolation in rhesus monkey kidney cells (Quidel, San Diego, California) along with WI-38 human embryonic lung fibroblasts (Quidel, San Diego, California). Cell lines with cytopathic effects are stained with murine monoclonal antibodies against RSV (D3 UltraTM DFA Respiratory Virus Screening and ID Kit, Quidel, San Diego, California). Samples submitted from patients in the ICU undergo multiplex PCR testing, but not culture.

### RNA extraction

Total nucleic acid was extracted from 250 μl of the supernatant of each RSV-positive cell-culture or primary sample using the NucliSens easyMAG automated extraction system (bioMerieux, Montreal, Canada) according to the manufacturer’s instructions.

### Real-time RT-PCR

Identification of RSV-A and B groups was conducted by targeting the nucleocapsid gene with a modified version of a previously published protocol^49^ on the ABI 7500 FAST platform (Applied Biosystems, California).

### Sequencing

A 900bp fragment of the G gene of RSV-A positive samples was further amplified with the Qiagen OneStep RT-PCR kit as previously described^50^. The second hypervariable G-gene region sequences (330bp: nt 5323–5652, corresponding to amino acid positions 212 to 321) of all RSV-A sequences obtained in this study (2011–2012) have been submitted to GenBank (accession numbers: KP321974-KP322010, KR871317-KR871349).

### RSV-A ON1 G-gene nucleotide sequences used in this study

All available second hypervariable region sequences (330bp: nt5323–5652) of G-gene (n = 483; Set G330) from all 20 countries that have submitted ON1 sequences to NCBI’s GenBank to date (spanning 2010 to 2014) were collected. Further, to understand the impact of multiple identical sequences derived from the same country on the estimates, we used 293 sequences (Set G330R) among the 483 global G-gene sequences after the removal of identical sequences originating in the same country (Set G330R). We also used 330bp sequences from Ontario, Canada (n = 60; Set ON) from our current and previous studies covering two RSV seasons (2010–2012)^11^. A 696bp region (4957–5652bp) of G-gene (n = 281; Set G696) from global sequences from 2010–2014 seasons were also collected to assess the impact of sequence length on molecular analyses. Set G696 sequences were available from 15 countries only. All non-Ontario sequences used in this study were obtained from GenBank (as of 14 November 2014; Fig. 2 and Table S1). BioEdit 7.2.5 was used for raw sequence analysis and curation^51^. MAFFT, a multiple sequence alignment server was used for the alignment of sequences^52^. Our global sequence datasets include Ontario sequences (n = 60).

### Phylogenetic analyses

Molecular Evolutionary Genetics Analysis (MEGA) version 6.0^53^ used to construct Neighbor Joining (NJ) and Maximum Likelihood (ML) phylogenetic trees and the robustness of the phylogenetic clusters was tested by bootstrapping with 1,000 iterations. The Ontario (set ON) phylogenetic tree was prepared by NJ algorithm using the Maximum Composite Likelihood (MCL) approach. ML trees with the four different RSV-A ON1 G-gene sequence Sets, G330, G330R, G696 and ON were constructed to estimate tMRCA by Path-O-Gen^27^, which uses regression of the root-to-tip distances from ML trees. The ML trees of the global (Set G330) and local (Set ON) were used for investigating the evolutionary pressure by Phylogenetic Analysis by Maximum Likelihood (PAML) program version 4.4^29^. We considered sampling collection date to classify the RSV-A ON1 lineages as there is uncertainty on the exact origin of RSV-A ON1 and lineage clusters were identified following its phylogeny and p-distances using pre-established nomenclature^54^.

### Phylodynamic analysis

We used Markov Chain Monte Carlo (MCMC) method implemented in Bayesian Evolutionary Analysis by Sampling Trees (BEAST v1.8.0)^34^ program to simulate phylogenies and estimates nucleotide substitution rate, tMRCA and growth rate (r) of the RSV-ON1 for time-stamped global (Sets G330, G330R and G696), and local (ON) G-gene sequences. Parametric demographic models (logistic growth and exponential growth) were used to yield tMRCA, and growth rate (r) of viral populations^34^,^55^. The substitution model HKY85 + gamma was used to estimate the rate of evolution. We used uncorrelated relaxed clock models that assume heterogeneous substitution rates across phylogenetic branches^56^.

Visual inspection of Bayesian sampled parameter estimates was conducted using Tracer v1.6 ensuring effective sampling size (ESS) of all parameters was ≥200. The uncertainty in the estimates is indicated by 95% Bayesian Credible Interval (BCI) values. Tree Annotator v1.8.0 was used to summarize the information in a sample of trees by choosing the tree with the maximum product of posterior probabilities. The Bayesian Maximum Clade Credibility (MCC) phylogeny annotated with divergence time, lineages, and evolutionary rate summaries was used as a representation of the evolutionary history of RSV-A ON1 and phylogeny visualized using FigTree v1.4.2 (http://tree.bio.ed.ac.uk/software/figtree/). We also compared Bayesian MCMC derived tMRCA with Path-O-Gen^27^.

### The basic reproductive number (*R*_*0*_) estimate using RSV-A ON1 G-gene sequences

The population growth rates (r, in years) of RSV-A ON1 approximated from the exponential growth and logistic growth models can be used to infer the epidemiological quantity, *R*_*0*_. *R*_*0*_ is the basic reproductive number (infectivity) of a pathogen. It can be defined as the average number of secondary infections caused by each index patient in a totally susceptible population^43^. If *R*_*0*_ is <1 each infected cases produces, on average, less than one new infected case and it is therefore predicted that the infection (or pathogen) will be cleared from the population. If *R*_*0*_ is >1 the pathogen has ability to propagate and increase among susceptible populations by producing more than one new infected case from each source case. *R*_*0*_ can be obtained either by the BEAST Tutorial [*R*_*0*_ = (1 + r/b)^a, where r is growth rate, a and b are gamma distribution parameters]^57^ and/or Pybus *et al.* [*R*_*0*_* = *rD + 1, where r is growth rate, and D is average duration of infectiousness]^58^. We used *R*_*0*_* = *(1 + r/b)^a. To calculate parameters a and b, we used mean generation time distribution or mean serial interval of RSV (μ = 7 days from Crowcroft *et al.*^28^) and standard deviation (σ) assumed to be 3.5 days.

### Selection pressure analysis

The program PAML 4.4 incorporates different codon-based substitution models that account for variable ω (non-synonymous/synonymous ratio, dN/dS) for each codon site^29^. CODEML program of PAML was used to understand the selection pressure at each codon site of ON1 genotype. We ran CODEML analyses with global RSV-A ON1 (Set G330) and Ontario RSV-A ON1 (Set ON) sequences (ON67-1210A (accession number: JN257693) was used as reference strain) and ML trees respectively.

Four different codon substitution models that account for neutral (M1a and M7) and positive (M2a and M8) selection were used in the analysis. The likelihood ratio tests (LRT) between nested models (M1a vs. M2a and M7 vs. M8) were conducted by comparing twice the difference in log-likelihood values (2Δ*l*) against a chi-square distribution with two degrees of freedom (d.f.) equal to the difference in the number of parameters between models^29^. If the LRT is significant (p < 0.0001), positive selection (ω = dN/dS ratio) is inferred. Bayes Empirical Bayes (BEB) approach (implemented in CODEML) was used to calculate the posterior probabilities (that takes sampling errors into account) of the inferred positively selected sites^59^. Sites with high posterior probabilities (PP) coming from the class with ω > 1 (P > 95%) are inferred to be under positive selection.

### Statistical Analysis

SPSS PASW v.18 (SPSS Inc., Chicago, Il) software was used to perform statistical analysis. Group comparisons were performed using chi-square exact test for categorical variables. P-values < 0.05 were considered statistically significant.

## Additional Information

**How to cite this article**: Duvvuri, V. R. *et al.* Genetic diversity and evolutionary insights of respiratory syncytial virus A ON1 genotype: global and local transmission dynamics. *Sci. Rep.* **5**, 14268; doi: 10.1038/srep14268 (2015).

## Supplementary Material

Supplementary Information

## Figures and Tables

**Table 1:** Population demographics of RSV positive individuals in Ontario (August 2011 to August 2012).

	RSV Positive	ON1	RSV-A^a^	RSV-B	p-value
	n = 406	n = 51	n = 319	n = 36	
Demographic Characteristics					
Age in years, median, IQR^b^	0,2	0,2	0,3	1,2	
Age group in years, n (%)					
[<1]	300 (73.9)	30 (58.8)	193 (60.5)	17 (47.2)	
[1–4]	48 (11.8)	13 (25.5)	83 (26.0)	12 (33.3)	
[5–19]	6 (1.5)	0 (0.0)	5 (1.6)	1 (2.8)	
[20–64]	23 (5.7)	2 (3.9)	18 (5.6)	3 (8.3)	
[65 + ]	29 (7.1)	6 (11.8)	20 (6.3)	3 (8.3)	
Gender, n (%)^c^					
Male	214 (52.7)	21 (41.2)	155 (48.5)	19 (52.7)	**p = 0.0097**
Female	192 (47.3)	30 (58.8)	100 (51.4)	17 (47.2)	
Setting, n (%)^d^					**p = 0.99**
Community	73 (17.9)	9 (12.3)	51 (70.0)	13 (17.8)	
Emergency Room	130 (32)	17 (13.1)	100 (76.9)	13 (10.0)	
Hospitalized (Non-ICU)	167 (41.1)	20 (12.0)	137 (82.0)	10 (6.0)	
ICU	36 (8.8)	5 (13.9)	31 (86.1)	0 (0.0)	

^a^Comprises RSV-A NA1 (n = 181), NA2 (n = 135), and GA5 (n = 3).

^b^IQR, interquartile range.

^c^Comparing males and females with RSV-A ON1. ^d^Comparing ON1 and Non-ON1 RSV-A and -B by patient setting X^2^ = 0.1449, p = 0.99.

**Table 2:** Estimated mean evolutionary rate, time of most recent common ancestor (tMRCA), and basic reproduction number (*R*_*0*_) of the analyzed global sequences of RSV-A ON1.

Model (Dataset)	Mean tMRCA (95% BCI)	Mean evolutionary rates(x 10^−3^) (95% BCI)	*R*_*0*_ (95% BCI)
Global			
Exponential growth (Set G330)	2008.08 (2006.46–2009.42)	4.1 (3.1–5.0)	1.017 (1.012–1.022)
Logistic growth (Set G330)	2007.78(2005.87–2009.30)	4.02 (3.04–5.04)	1.013 (1.008–1.026)
Exponential growth (Set G330R)	2008.81 (2007.40–2009.93)	4.12 (2.3–5.4)	1.032 (1.023–1.047)
Logistic growth (Set G330R)	2008.72 (2007.35–2009.79)	4.0 (2.5–5.03)	1.013 (1.008–1.018)
Exponential growth (Set G696)	2007.77 (2005.88–2009.40)	2.4 (1.8–3.07)	1.013 (1.008–1.026)
Logistic growth (Set G696)	2007.85 (2005.90–2009.45)	2.3 (1.8–2.9)	1.011 (1.002–1.021)
ML Tree (Set G330)^27^	2008.95	3.4	NA
ML Tree (Set G330R)^27^	2008.72	2.5	NA
ML Tree (Set G696)^27^	2007.67	1.9	NA
Ontario, Canada			
Exponential growth (Set ON)	2009.70 (2007.98–2010.53)	3.12(1.0–5.6)	1.03 (1.007–1.07)
Logistic growth (Set ON)	2009.46 (2007.37–2010.51)	3.58(1.07–6.6)	1.01 (1.0–1.03)
ML Tree (Set ON)^27^	2009.59	3.13	NA

Path-O-Gen^27^ used. NA: Not applicable

**Table 3:** Comparative mean estimates of global RSV-A ON1 time of most recent common ancestor (tMRCA).

Reference	Program Used	TreeMethod	ON1 G-GeneSequences Used	Probable tMRCA (95% BCI)
This study	BEAST^34^	MCC Tree	Set G330	2008.08 (2006.46–2009.42)
	BEAST^34^	MCC Tree	Set G330R	2008.81 (2007.40–2009.93)
	BEAST^34^	MCC Tree	Set G696	2007.77 (2005.88–2009.40)
This study	Path-O-Gen^27^	ML Tree	Set G330	2008.95
	Path-O-Gen^27^	ML Tree	Set G330R	2008.72
	Path-O-Gen^27^	ML Tree	Set G696	2007.67
Agoti *et al.*^14^	BEAST^34^	MCC Tree	65 sequencesfrom 7 Countries(333nt)	2009.12 (2004.26–2012.10)
Agoti *et al.*^14^	Path-O-Gen^27^	ML Tree	65 sequencesfrom 7 Countries(333nt)	2008.8
Ren *et al.*^21^	Path-O-Gen^27^	ML Tree	55 sequencesfrom 10 Countries(2^nd^ hypervariable region)	2010.8
Hirano *et al.*^12^	BEAST^34^	MCC Tree	93 sequencesfrom 11 Countries(2^nd^ hypervariable region)	2005 (2000–2010)

Set G330: All available second hypervariable region of G-gene sequences (330bp) from all 20 countries that have submitted ON1 sequences to NCBI’s GenBank. Set G330R: After the removal of identical sequences from Set G330 originating in the same country. Set G696: All available 696bp length G-gene sequences from all 15 countries that have submitted ON1 sequences to NCBI’s GenBank.
