# Supplementary material for: Genetic diversity and evolutionary insights of respiratory syncytial virus A ON1 genotype: global and local transmission dynamics
Source: Sci Rep. 2015 Sep 30;5:14268. doi: 10.1038/srep14268 (PMC4588507; doi:10.1038/srep14268)
Supplement: Supplementary Information [file srep14268-s1.pdf]

Supplementary Material

Title

Genetic diversity and evolutionary insights of respiratory syncytial virus A ON1 genotype: global and local transmission dynamics.

Authors:

Venkata R. Duvvuri[1,2#,*], Andrea Granados[1,3#], Paul Rosenfeld[1], Justin Bahl[4], Alireza Eshaghi[1], Jonathan B. Gubbay[1,3,5,6*]

# These authors contributed equally to this work.

Affiliations

[1] Public Health Ontario, Toronto, Ontario, Canada

[2] University of Waterloo, Waterloo, Ontario, Canada (MPH student)

[3] University of Toronto, Ontario, Canada

[4] Center for Infectious Diseases, The University of Texas School of Public Health, Houston, Texas, United States of America.

[5] Mount Sinai Hospital, Toronto, Ontario, Canada

[6] The Hospital for Sick Children, Toronto, Ontario, Canada

*Corresponding authors

Dr. Jonathan Gubbay
Medical Microbiologist
Public Health Ontario Laboratory
Ontario Agency for Health Protection and Promotion (OAHPP)
81 Resources Rd,
Toronto, ON M9P 3T1
jonathan.gubbay@oahpp.ca

Co-corresponding author:

Dr. Venkata R Duvvuri, venkata.duvvuri@oahpp.ca

Supplementary Material

Table S1. RSV-A ON1 G-gene sequences used in the study.

Country	Strain Identifier	Accession Number	G-gene Sequences (length 330bp): Number of sequences collected during season (October to September) 2010-2011	2011-2012	2012-2013	2013-2014	Total sequences	G-gene Sequences (length 696bp): Number of sequences collected during season (October to September) 2010-2011	2011-2012	2012-2013	2013-2014	Total sequences
Canada	ON, C, E, H, I	JN257693, JN257694, KP321974-KP322010, KR871317-KR871349	10	50			60	10	1			11
China	BJ, GZ	KC461212, KC461213, KC559442, KC559440, KM586845, KM586834			5		5			5		5
Croatia	HR	KF057865, KF057866, KF057867			2		2					0
Cuba	CU2011	KC754738, KC754743, KC677857-KC677877, KC283043, KC283044		8	12		20		8	12		20
Germany	HD, WUE	KJ710364-KJ710420, JX912355-JX912364		10	29		39					0
India	NIV	KF246639, KF246640, KC731482, KF246641, KF246638	1	4			5	1	4			5
Italy	RM, AN	JX988439 to JX988452, KC858199-KC858257		14	52		66					0
Japan	AH, AS, Chiba, Kanagawa, Yamaguchi, Yokohama	AB761609, AB761610, AB761611 AB754590, AB698559, AB808757-AB808780		13	16		29			4		4
Kenya	KEN	KF587911-KF588014		28	49		77		28	49		77
Korea	GM	JX627336		1			1		1			1
Latvia	LV	KF030154		1			1					0
Malaysia	MY	JX256871		1			1					0
Panama	PAN	KF300973-KF301018	13	9			22	13	9			22
Paraguay	Py	KM508823, KM508824,KM508825,KM508826			4		4			4		4
Peru	PER	KJ627264, KJ627347		1			1		1			1
Philippines	ONP, BPH	AB846656, AB846657, AB846655, AB846651, AB846652, AB846653			6		6			6		6
South Africa	SA, RXH	KC476744, KC476745, JX885730-JX885737		10			10		2			2
Spain	MAD, VH	KF915227-KF915265, KM402618-KM402671			25	53	78			25	53	78
Thailand	CU2011	KC342406, KC342413, KC342419, KC342434, KC342441	3	2			5	3	2			5
USA	LA2, TX	KJ890469-KJ890479, KM042382-KM042392, KJ672424-KJ672480		3	48		51			40		40
	**Total sequences used in the current study**		27	155	248	53	483	27	56	145	53	281
	**Total countries covered in the current analysis**		3	15	11	1	20	3	8	8	1	15

Supplementary Material

Table S2. Exact geographical area of collected specimens from each country.

Country (specimen collection period)	Province/City/Hospital/Surveillance systems	Reference
Canada (2010-2012)	Ontario	1
China (2012-2013)	Beijing, Chongqing, Shanghai	2-4
Croatia (2012-2013)	Exact location not available	Unpublished
Cuba (2011-2013)	Exact location not available	Unpublished
Cyprus (2012-2013)	Nicosia	5
Germany (2011-2013)	Bavaria, Heidelberg	6-7
India (2010-2012)	Pune	8
Italy (2011-2013)	Rome, Ancona	9
Japan (2011-2013)	Fukui, Chiba, Tochigi, Yamaguchi, Kanagawa	10-11
Kenya (2011-2012)	Kilifi	12
South Korea (2011-2012)	ARI-NET and KINRESS	13
Latvia (2011-2012)	Riga	14
Malaysia (2011-2012)	Kuala Lumpur	15
Panama (2010-2012)	Exact location not available	Unpublished[#]
Paraguay (2012-2013)	Exact location not available	Unpublished[#]
Peru (2011-2012)	Lima	Unpublished[#]
Philippines (2012-2013)	Palawan, Puerto Princesa	Unpublished[#]
South Africa (2011-2012)	Cape Town, Gauteng Province, KwaZulu-Natal Province, Mpumalanga Province, and North-West Province, Pretoria	16-17
Spain (2012-2014)	Catalonia	18
Thailand (2010-2011)	Bangkok and Khon Kaen provinces	19
USA (2011-2013)	Texas, Tennessee	20

[#] RSV-A ON1 G-gene sequences are directly submitted to NCBI Genbank. G-gene sequences from Cyprus are not available in NCBI Genbank.

Supplementary Material

References cited in Table S2.

1. Eshaghi, A. *et al*. Genetic variability of human respiratory syncytial virus A strains circulating in Ontario: A novel genotype with a 72 nucleotide G gene duplication. *PLOS One.* **7**, 1-10 (2012).
2. Cui, G. *et al*. Genetic variation in attachment glycoprotein genes of human respiratory syncytial virus subgroups A and B in children in recent five consecutive years. *PLOS One.* **8**, 1-12 (2013).
3. Liu, J. *et al*. Genetic variation of human respiratory syncytial virus among children with fever and respiratory symptoms in Shanghai, China, from 2009 to 2012. *Infect Genet Evol.* **27**, 131-6 (2014).
4. Ren, L. *et al.* The genetic variability of glycoproteins among respiratory syncytial virus subtype A in China between 2009 and 2013. *Infect Genet Evol.* **27**, 339-47 (2014).
5. Panayiotou, C. *et al*. Epidemiology of respiratory syncytial virus in children in Cyprus during three consecutive winter seasons (2010-2013): Age distribution, seasonality and association between prevalent genotypes and disease severity. *Epidemiol Infect.* **142**, 2406-11(2014).
6. Prifert, C., Streng, A., Krempl, C., Liese, J. & Weissbrich, B. Novel respiratory syncytial virus A genotype, Germany, 2011-2012. *Emerg Infect Dis.* **19**, 1029-30 (2013).
7. Tabatabai, J., Prifert, C., Pfeil, J., Grulich-Henn, J. & Schnitzler, P. Novel Respiratory Syncytial Virus (RSV) Genotype ON1 Predominates in Germany during Winter Season 2012–13. *PLOS One*. **9**, 1-9 (2014).
8. Choudhary, M., Anand, S., Wadhwa, B. & Chadha, M. Genetic variability of human respiratory syncytial virus in Pune, Western India. *Infect Genet Evol.* **20**, 369-77 (2013).
9. Pierangeli, A. *et al*. Rapid spread of the novel respiratory syncytial virus A ON1 genotype, central Italy, 2011 to 2013. *Euro Surveill.* **19**, 1-10 (2014).
10. Hirano, E. *et al*. Molecular evolution of human respiratory syncytial virus attachment glycoprotein (G) gene of new genotype ON1 and ancestor NA1. *Infect Genet Evol.* **28**, 183-91 (2014).
11. Tsukagoshi, H. *et al*. Genetic analysis of attachment glycoprotein (G) gene in new genotype ON1 of human respiratory syncytial virus detected in Japan. *Microbiol Immunol.* **57**, 655-9 (2013).
12. Agoti, C., Otieno, J., Gitahi, C., Cane, P. & Nokes, D. Rapid spread and diversification of respiratory syncytial virus genotype ON1, Kenya. *Emerg Infect Dis.* **20**, 950-9 (2014).
13. Kim, Y. *et al*. Rapid replacement of human respiratory syncytial virus A with the ON1 genotype having a 72 nucleotide duplication in G gene. *Infect Genet Evol*. **26**, 103-12 (2014).
14. Balmaks, R., Ribakova, I., Gardovska, D. & Kazaks, A. Molecular epidemiology of human respiratory syncytial virus over three consecutive seasons in Latvia. *J Med Virol.* **86**, 1971-82 (2014).

15. Khor, C., Sam, I., Hoopi, P. & Chan, Y. Displacement of predominant respiratory syncytial virus genotypes in Malaysia between 1989 and 2011. *Infect Genet Evol.* **14**, 357-60 (2013).
16. Valley Omar, Z., Muloiwa, R., Hu, N., Eley, B. & Hsiao, N. Novel respiratory syncytial virus subtype ON1 among children, Cape Town, South Africa, 2012. *Emerg Infect Dis.* **19**, 668-70 (2013).
17. Pretorius, M. *et al*. Replacement and positive evolution of subtype A and B respiratory syncytial virus G-protein genotypes from 1997-2012 in South Africa. *J Infect Dis.* **208**, S227-37 (2013).
18. Gimferrer, L. *et al*. Molecular epidemiology and molecular characterization of respiratory syncytial viruses at a tertiary care university hospital in Catalonia (Spain) during the 2013-2014 season. *J Clin Virol*. **66**, 27-32 (2015).
19. Auksornkitti, V. *et al*. Molecular characterization of human respiratory syncytial virus, 2010-2011: Identification of genotype ON1 and a new subgroup B genotype in Thailand. *Arch Virol.* **159**, 499-507 (2014).
20. Avadhanula, V. *et al*. Infection with Novel Respiratory Syncytial Virus Genotype Ontario (ON1) in Adult Hematopoietic Cell Transplant Recipients, Texas, 2011–2013. *J Infect Dis.* **211,** 582-9 (2015).

Table S3. Estimated mean evolutionary rates, and basic reproductive number (*R_0*) of RSV-A ON1 G-gene sequences[#] by country.

Country (number of sequences)	Coalescent Method	Mean Evolutionary Rate (substitutions/site/year)			*R_0*		
		$x10^{-3}$ Mean	95% BCI Lower	95% BCI Upper	Mean	95% BCI Lower	95% BCI Upper
Canada (n=60)	Exponential Growth	3.12	$1.0x10^{-3}$	$5.6x10^{-3}$	1.03	1.007	1.07
Germany (n=39)	Exponential Growth	5.5	$1.8x10^{-3}$	$8.9x10^{-3}$	1.04	1.008	1.08
Italy (n=66)	Exponential Growth	4.04	$1.4x10^{-4}$	$8.9x10^{-3}$	1.01	0.99	1.03
Japan (n=29)	Exponential Growth	6.6	$5.7x10^{-4}$	$1.5x10^{-2}$	1.01	1.0	1.03
Kenya (n=77)	Exponential Growth	2.23	$7.7x10^{-4}$	$3.9x10^{-3}$	1.003	1.004	1.01
Spain (n=78)	Exponential Growth	1.56	$4.3x10^{-4}$	$2.9x10^{-3}$	1.005	0.99	1.01
Panama (n=22)	Exponential Growth	2.97	$4.8x10^{-4}$	$5.6x10^{-3}$	1.02	1.0	1.04
USA (n=51)	Exponential Growth	1.9	$4.7x10^{-4}$	$3.7x10^{-3}$	1.01	1.0009	1.02

BCI: Bayesian credible intervals, *R_0*: Basic reproduction number

Second hypervariable region of G-gene (330bp length).

Table S4. R_0 values quantified for infectious diseases using genetic data and incidence data

**Pathogen**	**Genetic data**	**Number of sequences**	**R_0 from sequences (95% BCI)**	**R_0 range from incidence data**
Respiratory Syncytial Virus	G-gene (Set G330)	483	1.017 (1.012-1.022)[#]	1.2-2.1[1]
	G-gene (Set ON)	60	1.030 (1.007-1.07)[#]	
Pandemic 2009 H1N1	Hemagglutinin	23	1.2[2]	1.4 - 1.6[2]
Pandemic 2009 H1N1	whole genome	100[##]	1.12 (1.07 - 1.16)[3]	1.3 - 1.7[2-4]
Hepatitis C virus subtype 1a	E1, NS5	34, 59	2.93[5]	1.2[6]
Hepatitis C virus subtype 1b	E1, NS5	76, 53	2.67[5]	
Hepatitis C virus subtype 4	E1, NS5	22, 18	1.68[5]	
Hepatitis C virus subtype 6	E1, NS5	23, 40	1.21[5]	
2014 Ebola virus (EBOV)	whole genome	72	1.65 - 2.18[7]	1.51-2.53[8]
Middle East respiratory syndrome coronavirus (MERSCoV)	whole genome	10	1.21 (1.08 - 1.40)[9]	0.42-0.80[10]
Epizootic Rabies virus	concatenated G, N and non-coding regions	-	1.02-1.16[11]	1.66-2.33[12]
Methicillin-resistant Staphylococcus aureus (MRSA) - *SPA-type* t008	whole genome	30	1.24-1.34[13]	0.60-1.0[14]

Specific to RSV-A ON1 genotype from the current study

First two months of pandemic

Supplementary Material

References cited in Table S4.

1. Weber, A., Weber, M., & Milligan, P. Modeling epidemics caused by respiratory syncytial virus (RSV). *Math Biosci*. **172**, 95-113 (2001).
2. Fraser, C. *et al.* Pandemic potential of a strain of influenza A (H1N1): early findings. *Science* **324**:1557–1561 (2009).
3. Hedge, J., Lycett, S., & Rambaut, A. Real-time characterization of the molecular epidemiology of an influenza pandemic. *Biol Lett.* **9**, 20130331(2013).
4. White, L. *et al*. Estimation of the reproductive number and the serial interval in early phase of the influenza A/H1N1 pandemic in the USA. *Influenza Other Respir Viruses,* **3**, 267–276 (2009).
5. Pybus, O.G. *et al*. The epidemic behavior of the hepatitis C virus. *Science.* **292**, 2323-5 (2001).
6. Massad, E., Coutinho, F.A., Chaib, E., & Burattini, M.N. Cost-effectiveness analysis of a hypothetical hepatitis C vaccine compared to antiviral therapy. *Epidemiol Infect.* **137**, 241-9 (2009).
7. Stadler, T., Kuhnert, D., Rasmussen, D.A., & du Plessis, L. Insights into the Early Epidemic Spread of Ebola in Sierra Leone Provided by Viral Sequence Data. *PLoS Curr Outbreaks*. October 6, (2014).
8. Althaus, C.L. Estimating the Reproduction Number of Ebola Virus (EBOV) During the 2014 Outbreak in West Africa. *PLoS Curr Outbreaks*. September 2, (2014).
9. Cauchemez, S. *et al*. Middle East respiratory syndrome coronavirus: quantification of the extent of the epidemic, surveillance biases, and transmissibility. *Lancet Infect Dis,* **14**, 50-56 (2014).
10. Breban, R., Riou, J., & Fontanet, A. Interhuman transmissibility of Middle East respiratory syndrome coronavirus: estimation of pandemic risk. *Lancet*. **382**, 694-9 (2013).
11. Biek, R. *et al*. A high-resolution genetic signature of demographic and spatial expansion in epizootic rabies virus. *Proc Natl Acad Sci U S A*. **104,** 7993-8 (2007).
12. Coleman, P.G., Dye, C. Immunization coverage required to prevent outbreaks of dog rabies. *Vaccine*. **14**, 185-6 (1996).
13. Prosperi, M. *et al.* Molecular epidemiology of community-associated methicillin-resistant Staphylococcus aureus in the genomic era: a cross-sectional study. *Sci Rep* **3**, 1902 (2013).
14. Kajita, E., Okano, J. T., Bodine, E. N., Layne, S. P. & Blower, S. Modelling an outbreak of an emerging pathogen. *Nat Rev Microbiol*. **5**, 700–9 (2007).

Table S5. Parameter estimates, dN/dS, values of log-likelihood (*l*), positively selected sites, and likelihood ratio tests (LRT) in the C-terminal hypervariable region of G-gene of RSV-A ON1 genotype viruses circulating globally (2010-2014), and Ontario, Canada (2010-2012).

Model	Parameter estimates	Mean dN/dS	Log-likelihood (*l*)	Positively selected sites (PSS)	Model comparison (2Δ*l*, d.f, p)
**Global (set G330)**					
M1a	ω0=0.0, ω1 =1.00<br>p0= 0.26, (p1= 0.74)	0.73	-1371.540	not allowed	M1a vs. M2a : 9.26,<br>d.f = 2<br>p = 0.009
M2a	ω_0= 0.0, ω1=1.00<br>**ω2=2.93**<br>*p*0=0.28, p1=0.42<br>**(*p2=0.29*)**	1.29	-1366.909	Twenty PSS listed in Table S4	
M7	p= 0.013 q= 0.005	0.66	-1372.025	not allowed	M7 vs. M8 : 10.06,<br>d.f = 2,<br>p = 0.006
M8	p0= 0.75, **(*p1= 0.25*)**<br>p= 0.02, q=0.006<br>**ω=3.24**	1.30	-1366.991	Forty one listed in Table S4	
**Ontario (set ON)**					
M1a	ω0=0.0, ω1=1.00<br>*p*0=0.35 (*p*1=0.65)	0.65	-638.162	not allowed	M1a vs. M2a : 6.44,<br>d.f = 2<br>p = 0.03
M2a	ω_0= 0.33, ω1=1.0<br>**ω2=6.42**<br>*p*0=0.85, p1=0<br>**(*p2=0.15*)**	1.23	-634.939	Five PSS listed in Table S4	
M7	p=0.045, q=0.022	0.67	-638.171	not allowed	M7 vs. M8 : 6.45,<br>d.f = 2,<br>p = 0.02
M8	p_0 = 0.85, **(*p1=0.15*)**<br>p=16.2, q=32.05<br>**ω=6.43**	1.23	-634.942	Twelve PSS listed in Table S4	

Neutral models (M1a, and M7) were compared with their respective alternative (selection) models (M2a and M8), which allow $\omega > 1$. Model comparison can be calculated using $2\Delta l = 2\ (l_1 - l_0))$, where l_1 = LRT of alternative model; and l_0 = LRT of null model. Proportion of PSS and their corresponding ω-values in M2a and M8 models are in bolded italics.ON67-1210A (accession number: JN257693) was used as reference strain in both analyses.

Supplementary Material

Table S6. List of positively selected sites predicted by PAML (reference strain: ON67-1210A)

Type of dataset	CODEML model (AA[#])	PSS using Bayes Empirical Bayes (BEB) analysis		
		**p>50% to p<70% (mean dN/dS ± S.E.)**	**p>70% to p<90% (mean dN/dS ± S.E.)**	**p>90% (mean dN/dS ± S.E)**
**Ontario (set ON)**	M2a (5)	Y273 (3.9 ± 3.4)	T306 (5.8 ± 3.1)	V303 (6.6 ± 2.6)
		Y280 (3.8 ± 3.4)		
		L310 (3.7 ± 3.2)		
	M8 (12)	V225 (3.5 ± 3.1)	T306 (5.6 ± 2.9)	V303 (6.2 ± 2.6)
		T241 (3.4 ± 3.1)		
		Y273 (3.9 ± 3.2)		
		L274 (3.7 ± 3.0)		
		Y280 (3.8 ± 3.2)		
		L289 (3.5 ± 3.1)		
		Y304 (3.4 ± 3.1)		
		L310 (4.0 ± 3.0)		
		Q312 (3.4 ± 3.1)		
		T319 (3.4 ± 3.1)		
**Global (set G330)**	M2a (20)	L226 (2.1 ±1.3)	G232 (2.7 ±1.2)	V225 (3.3 ±1.4)
		T231 (2.1 ±1.3)	L247 (3.1 ±1.5)	V303 (3.2 ± 1.4)
		T245 (2.3 ±1.5)	H266 (2.6±1.4)	N318 (3.2 ± 1.4)
		T246 (2.0 ±1.3)	L274 (2.6 ±1.2)	
		T248 (2.5 ±1.3)	H290 (2.6±1.4)	
		N250 (2.2 ±1.2)	T319 (2.8 ±1.4)	
		E262 (2.4 ±1.3)		
		Y273 (2.2 ±1.4)		
		L289 (2.0 ±1.3)		
		T306 (2.0 ±1.3)		
		L310 (2.2 ±1.1)		
	M8 (41)	P217 (1.8 ± 1.1)	T245 (2.3 ± 1.2)	V225 (2.8 ± 0.9)
		K223 (1.7 ± 1.1)	T249 (2.4 ± 1.1)	G232 (2.7 ± 0.9)
		L226 (2.0 ± 1.2)	N251 (2.3 ± 1.1)	L247 (2.7 ± 1.0)
		T231 (2.1 ± 1.2)	E262 (2.5 ± 1.1)	L274 (2.6 ± 0.9)
		K233 (1.7 ± 1.1)	H266 (2.5 ± 1.1)	Y304 (2.8 ± 0.9)
		T235 (1.7 ± 1.1)	G272 (2.5 ± 1.1)	N318 (2.8 ± 0.9)
		T246 (2.1 ± 1.2)	Y273 (2.2 ± 1.1)	
		L248 (2.0 ± 1.2)	H290 (2.5 ± 1.1)	
		S250 (1.9 ± 1.2)	L310 (2.3 ± 1.0)	
		H258 (1.9 ± 1.2)	T319 (2.6 ± 1.0)	
		S260 (1.9 ± 1.2)		
		S275 (1.8 ± 1.0)		
		S283 (1.9 ± 1.2)		
		L289 (2.0 ± 1.1)		
		T292 (1.7 ± 1.1)		
		T293 (1.8 ± 1.1)		
		S294 (1.9 ± 1.2)		
		G296 (1.9 ± 1.1)		
		L298 (1.6 ± 1.0)		
		T306 (2.0 ± 1.1)		
		S307 (1.9 ± 1.1)		
		E308 (1.7 ± 1.1)		
		S313 (1.8 ± 1.1)		
		L314 (1.8 ± 1.1)		
		S317 (1.9 ± 1.1)		

PSS: Positively selected sites, S.E.: Square root of the variance.

#AA: Total number of amino acids with posterior probability greater than 50%

ON67-1210A (accession number: JN257693) was used as reference strain in both analyses.
